# High expression of thymosin beta 10 predicts poor prognosis for hepatocellular carcinoma after hepatectomy

**DOI:** 10.1186/1477-7819-12-226

**Published:** 2014-07-18

**Authors:** Haoyuan Wang, Shanshan Jiang, Yaojun Zhang, Ke Pan, Jianchuan Xia, Minshan Chen

**Affiliations:** 1 Department of Hepatobiliary Surgery, Cancer Center of Sun Yat-Sen University, 651 Dongfeng Road East, Guangzhou 510060, China; 2 State Key Laboratory of Oncology in South China, Collaborative Innovation Center for Cancer Medicine, Sun Yat-sen University Cancer Center, Guangzhou 510060, China

**Keywords:** thymosin beta 10, hepatocellular carcinoma, hepatectomy, prognosis, prognostic factor

## Abstract

**Background:**

Thymosin beta 10 (Tbeta10) overexpression has been reported in a variety of human cancers. However, the role of Tbeta10 in hepatocellular carcinoma (HCC) remains unclear. The aim of the present study was to analyze Tbeta10 expression in tumor and matched non-tumorous tissues, and to assess its prognostic significance for HCC after hepatectomy.

**Methods:**

The level of Tbeta10 mRNA and protein in tumor and matched non-tumorous tissues was evaluated in 26 fresh HCC cases by reverse transcription-polymerase chain reaction (RT-PCR) and western blot. Additionally, Tbeta10 protein expression in 196 HCC was analyzed by immunohistochemistry (IHC) and correlated with clinicopathological characteristics and survival.

**Results:**

Results from RT-PCR and western blot analysis show that the levels of Tbeta10 mRNA and protein were significantly higher in tumor tissues of HCC, compared to that in matched non-tumorous tissues (*P =* 0.01 and *P <*0.001, respectively). IHC staining showed that high expression of Tbeta10 was detected in 58.2% (114/196) of HCC cases. High expression of Tbeta10 was significantly associated with advanced TNM stage (*P* <0.001). Survival analysis demonstrated that high Tbeta10 was related to shorter overall survival (OS) (*P =* 0.000) and disease-free survival (DFS) (*P =* 0.000). Multivariate analysis showed that high expression of Tbeta10 was an independent prognostic factor for both OS (*P =* 0.001, HR = 4.135, 95% CI: 2.603 to 6.569) and DFS (*P =* 0.001, HR = 2.021, 95% CI: 1.442 to 2.832). Subgroup analysis revealed that high expression of Tbeta10 predicts poorer survival for early and advanced stage.

**Conclusions:**

Tbeta10 protein abnormal expression might contribute to the malignant progression of HCC. High expression of Tbeta10 predicts poor prognosis in patients with HCC after hepatectomy.

## Background

Hepatocellular carcinoma (HCC) is the fifth most common cancer and the third leading cause of cancer mortality worldwide [1]. Liver resection remains the curative option for HCC with a 5-year overall survival (OS) rate of 50 to 70% after curative hepatectomy, but the postoperative recurrence rate remains as high as 70 to 83.7% [2]. Unfortunately, at present, there are no accurate prognostic factors to predict prognosis of HCC patients on the basis of commonly used clinicopathological characteristics [3]. Therefore, it is critical to identify more effective prognostic factors for patients with HCC after hepatectomy.

Thymosin beta 10 (Tbeta10) is a naturally occurring peptide that was first isolated, along with other lymphocytopoietic factors, in 1966 by Goldstein *et al.* from the calf thymus [4]. Tbeta10 is a member of the β-thymosin family, which is widely distributed in many tissues, with proven biological activities as an actin-sequestering protein involved in cell motility. In recent years, Tbeta10 has been proposed as a multifunctional regenerative peptide [5] that is involved in many critical biological activities including cell proliferation, anti-apoptosis and angiogenesis [6-8]. In cancer study, the role of Tbeta10 is controversial. High levels of Tbeta10 expression have been found in the metastatic tumor of thyroid [9,10], and cutaneous malignancy [11], while low levels of Tbeta10 expression have been associated with metastatic cervical carcinoma [12]. More recently, the expression features of Tbeta10 and Tbeta4 was reported by Theunissen *et al*. [13], but Tbeta10’s relationship with clinicopathological characteristics and survival are still unknown. Thus, the aim of the present study was to evaluate the expression of Tbeta10 in tumor tissues of HCC and to assess its prognostic significance in HCC patients after hepatectomy.

## Methods

### Ethics statement

The research was approved by the institutional review board (IRB) of Sun Yat-sen University Cancer Center, and written informed consent was obtained from each patient involved in the study.

### Patients and tumor tissue samples

To detect the mRNA and protein level of Tbeta10 in HCC, fresh tumor and the matched adjacent non-tumorous tissues were collected from 26 patients with HCC who underwent hepatectomy between October 2012 and December 2012 in our department, the Department of Hepatobiliary Surgery, Cancer Center of Sun Yat-Sen University (Guangzhou, China).

A cohort of 196 consecutive patients who received hepatectomy for HCC in our department from January 2004 to December 2006 was enrolled. The including criteria for the present study were (1) no previous treatment for HCC before surgery, (2) histologic confirmation of HCC, (3) R0 resection, (4) no lymph node or extrahepatic metastasis, and (5) a follow-up period of ≥3.0 months.

The main clinical and pathological variables of all patients were described in detail in Table 1. In brief, there were 162 male and 34 female patients, with a median age of 47-years old (mean ± SD: 47.1 ± 12.3, range: 15 to 78). Tumor size ranged from 1.3 cm to 24.0 cm (mean ± SD: 7.3 ± 4.0), 80 patients (40.8%) had a tumor ≤5.0 cm, and 116 (59.2%) had a tumor >5.0 cm. A total of 163 patients (83.2%) had a single tumor, and 33 (16.8%) had 2 to 3 tumors. Of the cohort, 173 patients (88.3%) had hepatitis B virus (HBV) infection, and only 2 patients had hepatitis C virus (HCV) infection. According to the 7th edition tumor-node-metastasis (TNM) classification of the American Joint Committee on Cancer (AJCC) [14], 129 patients (65.8%) had stage I disease, 17 (8.7%) had stage II disease, and 50 (25.5%) had stage III disease (Table 1).

**Table 1:** Correlations between Tbeta10 expression and clinicopathological features of 196 patients with hepatocellular carcinoma

**Variables**	**All patients (n = 196)**	**Low Tbeta10 (n = 82)**	**High Tbeta10 (n = 114)**	***P *** **value**
Age (years)				
≤50	119		69	0.949
>50	77	32	45	
Gender				
Male	162	68	194	0.932
Female	34	14	20	
HBV infection				
Absent	23	10	13	0.865
Present	173	72	101	
AFP level				
≤25 ng/ml	61	30	31	0.161
>25 ng/ml	135	52	83	
Liver cirrhosis				
Absent	55	25	30	0.521
Present	141	57	84	
Tumor size (cm)				
≤5 cm	80	37	43	0.298
>5 cm	116	45	71	
Tumor number				
Single	163	73	90	0.063
2 to 3	33	9	24	
TNM stage				
I to II	146	72	74	**<0.001**
III	50	10	40	
Tumor differentiation				
I to II	100	44	56	0.531
III to IV	96	38	58	
Vascular invasion				
Absent	167	74	93	0.092
Present	29	8	21	

AFP, alpha fetoprotein; HBV, hepatitis B virus; Tbeta10, thymosin beta 10.

### RNA preparation and protein extraction

Total RNA was extracted by using Trizol solution (Invitrogen, Shanghai, China) according to manufacturer instructions. Total protein was extracted with RIPA buffer (Beyotime, Shanghai, China) according to the manufacturer’s instruction. RNA and protein samples were stored at -80°C until use.

### Real-time quantitative reverse transcription-polymerase chain reaction

To quantify the mRNA expression of Tbeta10, real-time PCR amplification was undertaken with a Bio-Rad CFX96 Real-time PCR System (Life Technologies, Carlsbad, CA, USA). The sequences of the sense and antisense primers were as follows: Tbeta10, sense: 5’-TGGCAGACAAACCAGACATGG-3’, and antisense: 5’-CGAAGAGGACGGGGGTAGG-3’ and GAPDH, sense: 5’-CTCCTCCTGTTCGACAGTCAGC-3’, antisense: 5’-CCCAATACGACCAAATCCGTT-3’. The reactions were performed in a final volume of 15 μL, consisting of 7.5 μL of 2× SYBR Green master mix (Invitrogen), 2-μL of each 5’- and 3’- primer (1.5 pmol/μL), 0.5 μL of the sample cDNA, and 5 μL water. The amplification was performed as follows: 95°C for 10 min, one cycle, followed by 95°C for 30 s and 60°C for 60 s, 45 cycles. The relative expression levels of thymosin-10 were normalized to that of the internal control gene, GAPDH. Data were analyzed using the comparative threshold cycle (2- -ΔΔCT) method.

### Western blotting

The HCC samples including tumor and non-tumorous tissues were lysed in RIPA lysis buffer. Lysates were harvested by centrifugation (12,000 rpm for 30 min at 4°C. Protein samples (30 μg) were resolved in 12% sodium dodecyl sulfate polyacrylamide gel for electrophoresis and transferred to a polyvinylidene difluoride (PVDF) membrane. After blocking nonspecific binding sites for 60 min with 8% nonfat milk, membranes were incubated overnight at 4°C with a rabbit antibody against Tbeta10 (1:1,000 dilution; Abcam, Cambridge, UK) or GAPDH (1:10,000 dilution; Abcam, Cambridge, UK). Membranes were washed four times with TRIS-buffered saline with Tween-20 for 10 min. After being washed, membranes were probed with HRP-conjugated secondary antibody and visualized using a chemiluminescent system (Cell Signaling Technology, Danvers, MA, USA). Band intensity was measured by Quantity One software (BioRad, Hercules, CA, USA).

### Immunohistochemistry

Isolated tumors were fixed in 10% neutral buffered formalin for 48 h and embedded in paraffin according to standard protocols. Sections (thickness, 2 μm) were deparaffinized and rehydrated in a graded series of 100%, 95%, 90%, 80% and 70% ethanol. For antigen retrieval, slides were boiled in EDTA (1 mM, pH 8.0) for 15 min in a microwave oven. Endogenous peroxidase activity was blocked in 3% H_2_O_2_ at room temperature for 10 min. Sections were then stained with anti-Tbeta10 (rabbit anti-Tbeta10 monoclonal antibody; 1:500 dilution; Abcam, Cambridge, UK) antibodies at 4°C overnight. After three washes in PBS, sections were incubated with horseradish peroxidase (HRP)-conjugated secondary antibody (Envision Detection kit, GK500705, Gene Tech, Shanghai, China) for 30 min at room temperature. After washing in PBS, antibody complexes were colored with 3,3’-diaminobenzidine (DAB ) and then counterstained with hematoxylin. Slides were dehydrated and evaluated.

### Immunohistochemistry evaluation

The specimens were analyzed by two pathologists who were blinded to the patients’ clinical outcomes. The total Tbeta10 immunostaining score was calculated as the sum of the positively stained tumor cells and staining intensity. Briefly, the percentage of positive staining was scored as ‘0’ (0 to 9%, negative), ‘1’ (10 to 25%, sporadic), ‘2’ (26 to 50%, focal), or ‘3’ (>50%, diffuse). The staining intensity was scored as ‘0’ (no staining), ‘1’ (weak staining), ‘2’ (moderate staining), or ‘3’ (strong staining). The total immunostaining score was calculated as the value of percent positivity score × staining intensity score, and ranged from 0 to 9. The expression level of Tbeta10 was defined as follows: ‘-’ (score 0 to 1), ‘+’ (2 to 3), ‘++’ (4 to 6) and ‘+++’ (>6). Based on their levels of Tbeta10 expression, patients were divided into two groups: low Tbeta10 (‘-’ and ‘+’) and high Tbeta10 (‘++’ and ‘+++’).

### Follow-up

Follow-up of patients included physical examination, routine laboratory testing, and contrast-enhanced abdominal computed tomography (CT) every 3 months in the first 2 years, every 6 months in the 3 to 5 years after surgery, and then every year thereafter. At each follow-up visit, liver function tests and AFP were determined. Chest radiography was done every 6 months to observe lung metastasis. If necessary, CT of the chest, bone scintigraphy and positron emission tomography (PET) were also performed for the diagnosis of metastasis and/or recurrence. The last follow-up date for patients still alive was November 2013.

Causes of death and sites of recurrence were determined from death certificates, medical interviews, and radiological findings. Overall survival (OS) was defined as the interval between the time of hepatectomy to death or to the last date of follow-up. Disease-free survival (DFS) time was between the time of hepatectomy and the time when recurrence was diagnosed or to the time of the last follow-up. The treatment for recurrent tumor was determined by our multidisciplinary team (MDT) including surgeons, oncologists, radiologists, gastroenterologists, and pathologists.

### Statistical analysis

The statistical analyses were performed using the SPSS 13.0 statistical software (SPSS Company, Chicago, Illinois, USA). Comparisons between two groups were done using Student’s t-test for continuous data and the Chi square test for categorical data. The correlation between the Tbeta10 expression and clinicopathologic characteristics was analyzed with the Chi square test. The OS and DFS were calculated by Kaplan-Meier method and compared by log-rank test. The prognostic varieties in predicting OS and DFS were assessed by multivariate Cox proportional hazards regression analysis. Results were given as mean ± S.D. All statistical tests were two-sided, and a significant difference was considered when *P <*0.05.

## Results

### Tbeta10 mRNA and protein expression in hepatocellular carcinoma tissues

**Figure 1:**
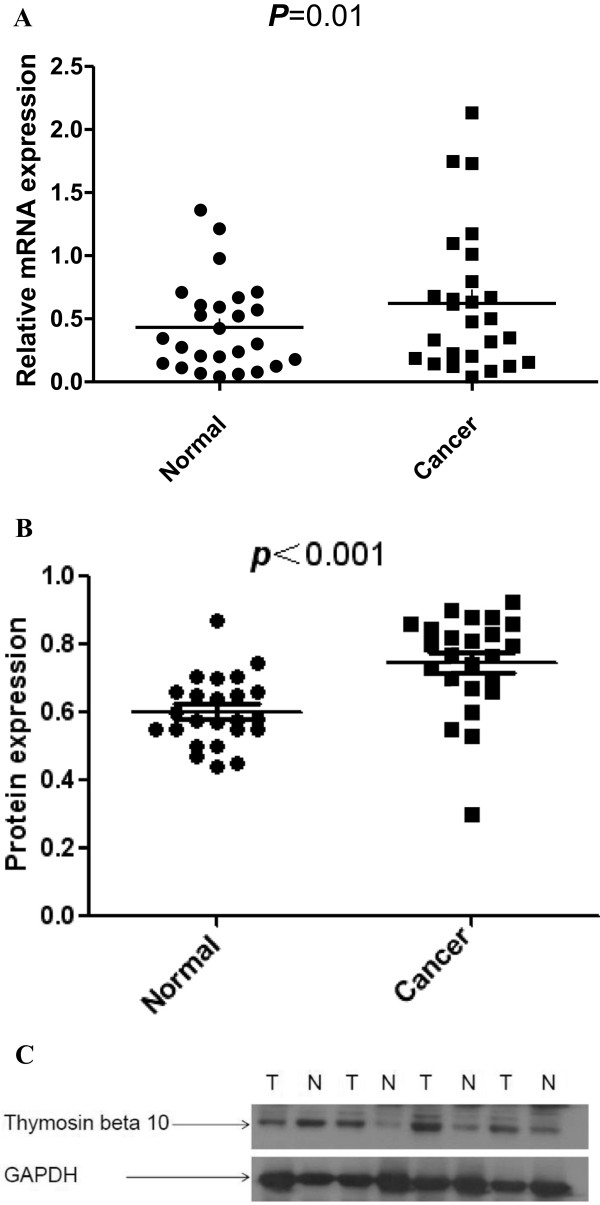
**The mRNA and protein expression of Tbeta10 was significantly higher in hepatocellular carcinoma (HCC) tumor than in matched non-tumorous tissues. (A)** The mRNA expression of Tbeta10 in human HCC specimens was evaluated by real-time quantitative PCR. The relative mRNA expression of Tbeta10 was significantly higher in 26 HCC tumor tissues than in matched non-tumorous tissues (*P =* 0.01). Horizontal lines represent the mean. **(B)** Relative Tbeta10 protein expression levels in 24 HCC tumor tissues and matched non-tumorous tissues (Tbeta10/GAPDH, *P* = 0.0005) were detected by western blot. Horizontal lines represent the mean. **(C)** Representative result of Tbeta10 protein expression in four paired HCC tumor tissues and the matched non-tumorous tissues (T, hepatocellular carcinoma tumor tissues; N, matched non-tumorous tissues).

The mRNA level of Tbeta10 was determined by real-time quantitative RT-PCR assays in 26 fresh tumor and the matched adjacent normal tissues. The Tbeta10 expression level was significantly higher in 19 (73.08%) tumor tissues compared to the adjacent non-tumorous tissues (*P* = 0.01, Figure 1A). The protein level of Tbeta10 was also evaluated by western blotting in 24 HCC tissues. As shown in Figure 1B, consistent with the results of the real-time quantitative RT-PCR, the protein expression of Tbeta10 was significantly higher in 18 (75%) tumor tissues, compared to that in adjacent non-tumorous tissue (*P <*0.001).

### Immunohistochemical analysis of Tbeta10 expression in hepatocellular carcinoma specimens and Tbeta10’s relationship with the clinicopathological parameters

**Figure 2:**
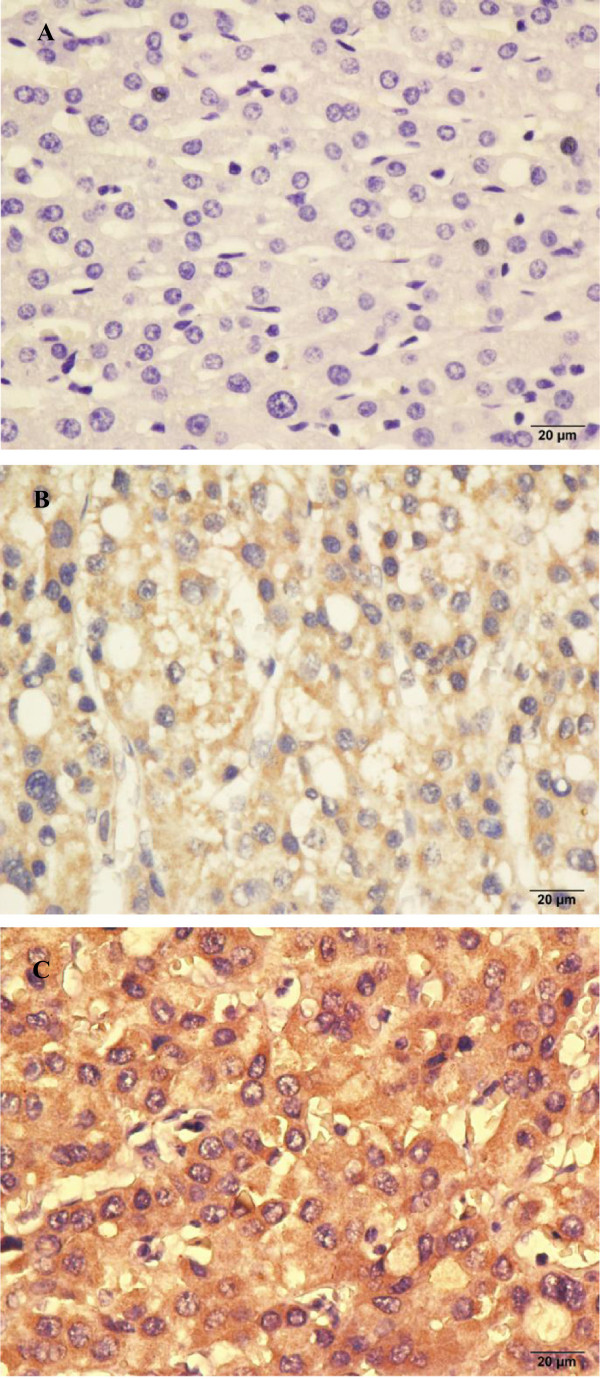
**Representative immunohistochemical staining of Tbeta10 in tumorous and non-tumorous tissues. (A)** Normal liver tissue distant from the tumor, scored Tbeta10 (-), **(B)** Low expression of Tbeta10 in non-tumorous tissue, scored Tbeta10 (‘-’ and ‘+’); **(C)** High expression of Tbeta10 in tumor tissue, scored Tbeta10 (‘++’ and ‘+++’). (**A-C** with 400 × magnification).

Tbeta10 expression was investigated in 196 HCC paraffin-embedded tissues of surgical specimens using IHC staining. As it was shown in Figure 2, Tbeta10 immunostaining was predominantly observed in the cytoplasm of carcinoma cell and rarely in nucleus. Among these 196 HCC cases, 114 (58.2%) showed high Tbeta10 expression, and 82 (41.8%) showed low Tbeta10 expression. The correlations between Tbeta10 expression level and clinicopathological features were shown in Table 1. Tbeta10 expression was significantly correlated with advanced TNM stage (*P <*0.001). However, no significant relationship was found between Tbeta10 expression and gender, age, HBV infection, alpha fetoprotein (AFP) level, underlying liver cirrhosis, tumor differentiation, tumor size, tumor number and vascular invasion (Table 1).

### The relationship of Tbeta10 expression and the survival of hepatocellular carcinoma patients

**Figure 3:**
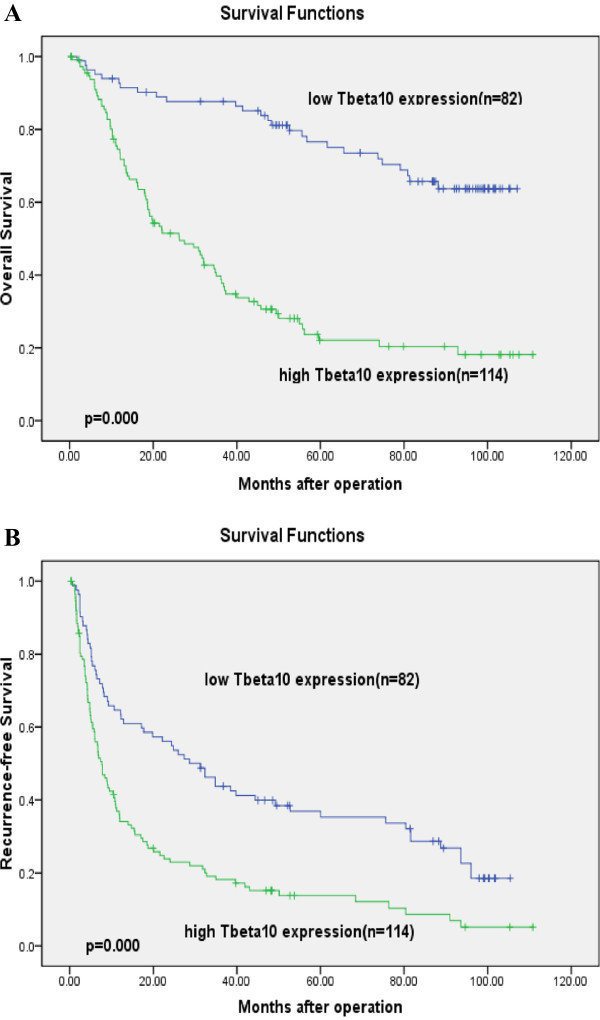
**Overall and recurrence-free survival curves of 196 hepatocellular carcinoma (HCC) cases after hepatectomy assessed by Kaplan-Meier analysis according to Tbeta10 expression.** Patients with high expression of Tbeta10 were significantly associated with poorer overall survival (**A**, *P* = 0.000) and disease-free survival (**B**, *P* = 0.000).

The mean follow-up period was 47.5 ± 35.0 months (range 3.0 to 110.7 months). At the end of follow-up, there were 108 deaths and 88 survivals. The 1, 3, and 5-year OS of the whole group was 82%, 60% and 46%, respectively. Univariate analysis showed that the OS was directly influenced by underlying liver cirrhosis (*P* = 0.026), tumor size (*P* = 0.024), tumor number (*P* = 0.042), TNM stage (*P <*0.001), tumor differentiation (*P* = 0.018), vascular invasion (*P* <0.001), and expression of Tbeta10 (*P <*0.001). Factors not significantly affecting OS included age, gender, HBV infection, and AFP level. Multivariate analysis showed that expression of Tbeta10 (*P* <0.001, HR = 4.135; 95% CI: 2.603-6.569), vascular invasion (*P* = 0.002, HR = 2.051; 95% CI: 1.288-3.264) and TNM stage (*P* = 0.018, HR =1.649; 95% CI: 1.091-2.492) were independent prognostic factors for OS (Table 2). The 1, 3, and 5-year OS for patients with Tbeta10 high expression was 75%, 40%, and 23%, respectively, and 93%, 88%, and 77%, respectively, for patients with Tbeta10 low expression (*P* <0.001, Figure 3A).

**Table 2:** Univariate and multivariate analyses of overall and disease-free survival for 196 hepatocellular carcinoma (HCC) patients

**Variables**	**Univariate analysis**	**Multivariate analysis**	
	***P *value**	**HR (95% CI)**	***P *value**
**Overall survival**			
Age (≤50 y versus >50 y)	0.429		
Gender (male versus female)	0.713		
HBV infection (absent versus present)	0.10		
AFP level (≤25 ng/ml versus >25 ng/ml)	0.234		
Liver cirrhosis (absent versus present)	**0.026**		
Tumor size (≤5 cm versus >5 cm)	**0.024**		
Tumor number (single versus multiple)	**0.042**		
TNM stage (I to II versus III)	**<0.001**	1.649 (1.091 to 2.492)	**0.018**
Tumor differentiation (I to II versus III to IV)	**0.018**		
Vascular invasion (present/absent)	**<0.001**	2.051 (1.288 to 3.264)	**0.002**
Tbeta10 (low versus high)	**<0.001**	4.135 (2.603 to 6.569)	**0.001**
**Disease-free survival**			
Age (≤55 y versus >55 y)	0.523		
Gender (male versus female)	0.515		
HBV infection (absent versus present)	**0.009**		
AFP level (≤25 ng/ml versus >25 ng/ml)	0.170		
Liver cirrhosis (absent versus present)	0.361		
Tumor size (≤5 cm versus >5 cm)	**0.006**	1.483 (1.047 to 2.100)	**0.027**
Tumor number (single versus multiple)	**0.018**		
TNM stage (I to II versus III)	**<0.001**	1.537 (1.060 to 2.228)	**0.023**
Tumor differentiation (I to II versus III to IV)	**0.003**		
Vascular invasion (present/absent)	**<0.001**	2.082 (1.378 to 3.145)	**0.001**
Tbeta10 (low versus high)	**<0.001**	2.021 (1.442 to 2.832)	**0.001**

AFP, alpha fetoprotein; HBV, hepatitis B virus; HR, hazard ratio; Tbeta10, thymosin beta 10.

The 1-, 3-, and 5-year DFS of the whole group was 48%, 29% and 24%, respectively. Univariate analysis showed that HBV (*P* = 0.009), tumor size (*P* = 0.006), tumor number (*P* = 0.018), TNM stage (*P* <0.001), tumor differentiation (*P* = 0.003), vascular invasion (*P* <0.001) and expression of Tbeta10 (*P* <0.001) were prognostic factors of DFS. Multivariate analysis indicated that expression of Tbeta10 (*P* = 0.001, HR = 2.021; 95% CI: 1.442 to 2.832), tumor size (*P* = 0.027, HR = 1.483; 95% CI: 1.047 to 2.100), vascular invasion (*P* = 0.001, HR = 2.082; 95% CI: 1.387 to 3.145) and TNM stage (*P* = 0.023, HR =1.537; 95% CI: 1.060 to 2.228) were independent prognostic factors of DFS (Table 2). The 1-, 3-, and 5-year DFS for patients with Tbeta10 high expression were 36%, 18%, and 14% respectively, and 65%, 44%, and 37% respectively, for patients with Tbeta10 low expression (*P* = 0.000, Figure 3B).

**Figure 4:**
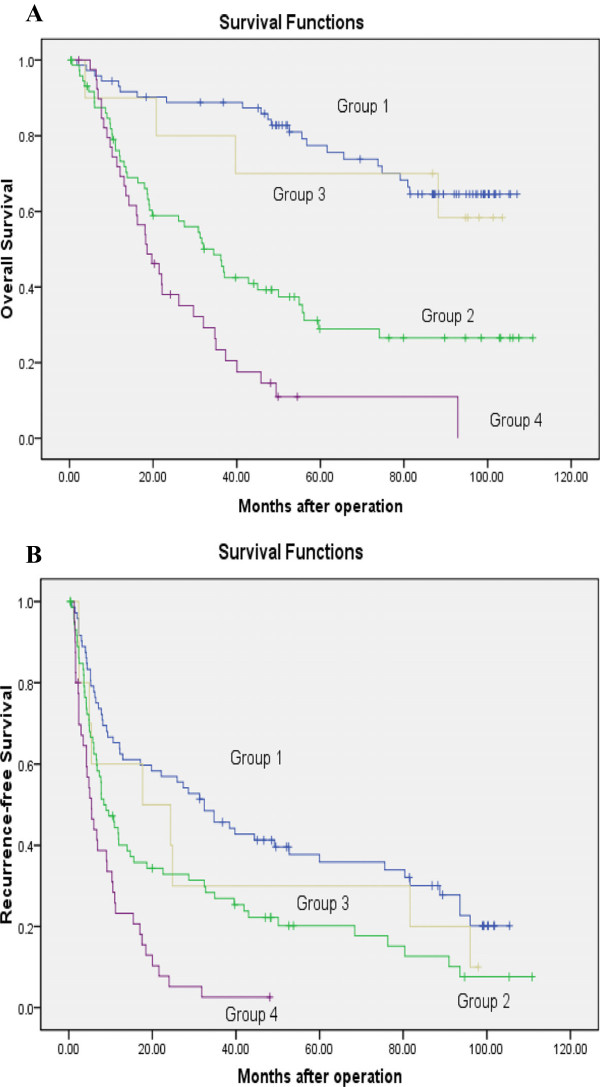
**Overall and disease-free survival curves assessed by Kaplan-Meier analysis according to Tbeta10 expression and TNM stage. (A)** Overall survival. High expression of Tbeta10 predicted poorer overall survival (OS) not only for early TNM stage (TNM stage I to II, group 1 versus group 2; *P* = 0.001), but also for advanced TNM stage (TNM stage III, group 3 versus group 4; *P* <0.001). **(B)** Disease-free survival. High expression of Tbeta10 predicts poor DFS for both early TNM stage (group 1 versus group 2; *P* = 0.003) and advanced TNM stage (group 3 versus group 4; *P* = 0.011). Group 1, early TNM stage/Tbeta10 low expression (n = 72); group 2, early TNM stage/Tbeta10 high expression (n = 74); group 3, advanced TNM stage/Tbeta10 low expression (n = 10); group 4, advanced TNM stage/Tbeta10 high expression (n = 40).

Furthermore, the correlation of Tbeta10 expression with survival was analyzed according to TNM stage. For 146 patients with TNM stage I and II disease after curative resection, the 1-, 3-, and 5-year OS for patients with Tbeta10 high expression was 76%, 48%, and 29%, respectively, and 93%, 89%, and 78%, respectively, for patients with Tbeta10 low expression (*P* <0.001), the corresponding DFS was 42%, 27%, and 20%, respectively, and 65%,46%, and 38%, respectively (*P* = 0.003). For 50 patients with TNM stage III disease, the 1-, 3-, and 5-year OS for patients with Tbeta10 high expression was 72%, 24%, and 11%, respectively, and 90%, 80%, and 70%, respectively, for patients with Tbeta10 low expression (*P* = 0.001), the corresponding DFS was 60%, 30%, and 30%, respectively, and 24%, 3%, and 3%, respectively (*P* = 0.011, Figure 4).

## Discussion

In the present study, we demonstrated that the expression of Tbeta10 was significantly higher in HCC tissue, compared to that in non-tumorous liver tissue, high expression of Tbeta10 was significantly associated with advanced TNM stage, and for the first time, we revealed that high expression of Tbeta10 predicted poorer survival for patients with HCC after hepatectomy, even in patients subgrouped according to TNM stage.

There has been increasing interest in the role of Tbeta10 in human cancers in recent years. Tbeta10 has been shown to be aberrantly expressed in many cancer types. IHC staining performed on HCC, human melanoma, renal cell carcinoma, gastric, breast, lung, and thyroid cancers showed strong positive staining of Tbeta10 specifically in tumor tissues compared with weak staining in the surrounding non-tumorous tissues [11,13,15-19]. Similarly, several reports have shown the correlation of Tbeta10 expression level with progression and metastasis as well as poor patient outcome [9,15]. Unlike its proposed oncogenic role, Tbeta10 has been found to be downregulated in human ovarian cancer [7], and low expression of Tbeta10 is associated with metastatic phenotype of cholangiocarcinoma *in vitro* and *in vivo*[20]. There are contradicting findings regarding Tbeta10 expression in ovarian and lung cancers [21-24]. In HCC the higher incidence of Tbeta10 IHC reactivity was found in tumor cells involved in stromal invasion, indicating a possible major role for Tbeta10 in HCC progression [13]. In the present study, we conducted RT-PCR, western blot and IHC to detect the expression of Tbeta10 in tumor and the matched adjacent non-tumorous tissues. Our results show that the expression of Tbeta10 was significantly higher in tumor tissues than that in non-tumorous tissues, which indicated that Tbeta10 might play an important part in the carcinogenesis of HCC.

Tbeta10, as a member of the β-thymosin family, is the main intracellular G actin-sequestering protein involved in cell proliferation, migration, and differentiation [6-8]. Many reports described the potential functional roles of Tbeta10 in human cancers. However, these functions are quite different among different types of cancers. Tbeta10 inhibits tumor growth, angiogenesis, migration, and invasion of ovarian cancer both in *in vitro* and *in vivo* studies by disrupting actin polymerization and by inhibiting Ras action [20,25]. In pancreatic cancer, Tbeta10 stimulates secretion of proinflammatory cytokines interleukin (IL-7) and IL-8, which may promote pancreatic cancer pathogenesis and progression [26]. Studies on thyroid carcinoma, melanoma, non-small cell lung cancer and some other malignant tumors [21,27,28] also show that Tbeta10 possesses tumor progression properties. In the present study, we demonstrated that high expression of Tbeta10 was associated with advanced TNM stage. It indicated that overexpression of Tbeta10 was associated with tumor growth and invasion in HCC.

Hepatectomy remains the most effective curative therapy and provides better survival outcomes for patients with HCC. Unfortunately, approximately 33% of HCC patients die within the first year even after curative surgery, mainly because of tumor recurrence and spread [29,30]. Currently, prognostic evaluation is mainly based on tumor stage and histopathologic observation such as tumor size, tumor number, and vascular invasion [1-3]. However, we found that although patients have modest tumor presentation, the prediction for patients’ overall and disease-free survival can be variable and inaccurate. Recent studies have suggested some factors, such as molecular and cellular characteristics of primary tumor, may improve our ability to prognosticate [4]. In the present study, the expression of Tbeta10 was revealed as an independent prognostic factor for both OS and RFS for patients with HCC after hepatectomy. The patients with high expression of Tbeta10 had a shorter OS and RFS. More importantly, subgroup analysis showed the expression of Tbeta10 was significantly associated with poor prognosis not only in patients with early TNM stage undergoing curative resection, but also in patients with advanced TNM stage undergoing palliative resection. It might indicate that Tbeta10 is one of the reliable clinical predictors for outcome of individual patients with HCC undergoing hepatectomy. However, prospective clinical studies are needed to confirm that.

There were some limitations in the present study that require comment. First, it was a retrospective analysis from a single institution with small sample size, and second patient population is biased due to high prevalence of HBV infection (88.3%). Whether these results can be applied to Western populations wherein HCV, and other etiologies of liver disease predominate, requires further study and comment. Therefore, a large-scale prospective validation study is needed to confirm the results.

## Conclusions

In conclusion, our study revealed that Tbeta10 is an independent prognostic factor for OS and DFS in patients with HCC after hepatectomy. High expression of Tbeta10 in tumor is correlated with advanced TNM stage. However these results, which are based on a Chinese cohort (mostly associated with HBV infections), should be further confirmed in other populations of patients with HCC.

## Abbreviations

AFP: alpha fetoprotein; CT: computed tomography; DFS: disease-free survival; HBV: hepatitis B virus; HCV: hepatitis C virus; HCC: hepatocellular carcinoma; IHC: immunohistochemistry; MDT: multidisciplinary team; OS: overall survival; PET: positron emission tomography; RT-PCR: reverse transcription-polymerase chain reaction.

## Competing interests

The authors declare that they have no competing interests.

## Authors’ contributions

HYW, SSJ, JCX and MSC are responsible for study design, experiments, data analysis and interpretation, and for drafting the manuscript. YJZ, PK, JCX and MSC participated in study design, data analysis and interpretation. All authors read and approved the final manuscript.
